# Mesenchymal Stem Cells in the Treatment of New Coronavirus Pandemic: A Novel Promising Therapeutic Approach

**DOI:** 10.34172/apb.2022.023

**Published:** 2021-03-27

**Authors:** Sara Razi, Zahra Molavi, Seyed Amir Mirmotalebisohi, Zahra Niknam, Marzieh Sameni, Vahid Niazi, Amirjafar Adibi, Mohsen Yazdani, Mohammad Mehdi Ranjbar, Hakimeh Zali

**Affiliations:** ^1^Proteomics Research Center, Shahid Beheshti University of Medical Science, Tehran, Iran.; ^2^Department of Biology, Science and Research Branch, Islamic Azad University, Tehran, Iran.; ^3^Student Research Committee, Department of Biotechnology, School of Advanced Technologies in Medicine, Shahid Beheshti University of Medical Sciences, Tehran, Iran.; ^4^Cellular and Molecular Biology Research Center, Shahid Beheshti University of Medical Sciences, Tehran, Iran.; ^5^Department of Tissue Engineering and Applied Cell Sciences, School of Advanced Technologies in Medicine, Shahid Beheshti University of Medical Sciences, Tehran, Iran.; ^6^Departments of Orthopedics, Faculty of Medicine, Shahid Beheshti University of Medical Sciences, Tehran, Iran.; ^7^Institute of Biochemistry and Biophysics, Tehran University, Tehran, Iran.; ^8^Razi Vaccine and Serum Research Institute, Agricultural Research, Education and Extension Organization, Karaj, Iran.

**Keywords:** COVID-19, SARS-CoV-2, Stem cell therapy, Mesenchymal stem cell, Cytokine storm

## Abstract

After severe acute respiratory syndrome (SARS) and Middle East respiratory syndrome (MERS) outbreaks, coronavirus disease 2019 (COVID-19) is the third coronavirus epidemic that soon turned into a pandemic. This virus causes acute respiratory syndrome in infected people. The mortality rate of SARS-CoV-2 infection will probably rise unless efficient treatments or vaccines are developed. The global funding and medical communities have started performing more than five hundred clinical examinations on a broad spectrum of repurposed drugs to acquire effective treatments. Besides, other novel treatment approaches have also recently emerged, including cellular host-directed therapies. They counteract the unwanted responses of the host immune system that led to the severe pathogenesis of SARS-CoV-2. This brief review focuses on mesenchymal stem cell (MSC) principles in treating the COVID-19. The US clinical trials database and the world health organization database for clinical trials have reported 82 clinical trials (altogether) exploring the effects of MSCs in COVID-19 treatment. MSCs also had better be tried for treating other pathogens worldwide. MSC treatment may have the potential to end the high mortality rate of COVID-19. Besides, it also limits the long-term inability of survivors.

## Introduction

Severe acute respiratory syndrome Coronavirus 2 (SARS-CoV-2) is a member of a large family of viruses called Coronaviridae that cause a various range of illnesses from the common cold to the more severe diseases, such as Middle East respiratory syndrome (MERS) and SARS. The infectious disease caused by SARS-CoV-2 has been named coronavirus disease 2019 (COVID-19).^1^ Coronaviruses were first discovered and studied in the 1960s.^2^ The virus was under vigorous investigations until the mid-1980s.^3^ The virus naturally spreads to birds and mammals; however, seven human-transmitted coronaviruses have been discovered. The most recent coronaviruses, the agent of SARS-CoV-2, became epidemic in 2019 in Wuhan, China. The new coronavirus soon spread worldwide and turned into a disastrous pandemic, leading to thousands of deaths.^2^

Symptoms of the new virus that cause COVID-19 usually start a few days after being infected. However, in certain people, the symptoms may appear later (from two to fourteen days after infection). According to statistics and research, symptoms include fever, dry cough, respiratory distress, fatigue, muscle aches, and even diarrhea (in 3.8% of cases). The average incubation period was four days. Turbidity, or Ground-glass opacity, was observed in 4.56% of chest scans of patients with COVID-19. Around nine percent of the patients with the non-severe form of the disease did not show any problems in their radiology or scintigraphy outcomes. Lymphocytopenia (decrease in circulating lymphocytes) was observed in a subset of patients at admission.^4^ The patients infected with SARS-CoV-2 may show no manifestations of the disease. However, many patients experience mild to severe symptoms that may lead to deadly pneumonia.^5^ The immune response is essential for the control and elimination of the SARS-CoV-2. Any defects in the immune system may lead to a severe form of the disease.^6^

Currently, little is known about the status of the innate immune system in COVID-19. The innate system is our first line of defense against SARS-CoV-2. Various case studies have shown that in some COVID-19 patients, the number of lymphocytes increases. Besides, neutrophil counts are shown to decrease in some patients with COVID-19. The lymphopenia is shown to be directly associated with the severity of the disease.^7^

Initially, the immune system needs to identify the virus. The immune system uses specific immune receptors, called pathogen-associated molecular pattern molecules (PAMPs), to detect the invaders. PAMP is the abbreviation for Pathogen-associated molecular pattern molecules. These receptors can detect the genetic material of various viruses. The genetic material of the SARS-CoV-2 is made of single-strand RNA. Identifying the viral genome leads to the activation of intracellular pathways that eventually lead to the production of interferon type 1 (IFN-I).^8^ IFN-I is the most effective intrinsic immune defense against the virus, and its successful increase leads to inhibition of virus replication and elimination in the early stages.^8,9^ SARS-CoV-2 suppresses this response by disrupting interferon production or messaging. This strategy is closely related to the severity of the disease. Following the suppression of this defense barrier, immune cells present at the site of entry of the virus produce increased levels of IFN-I to compensate, which infiltrates more macrophages and neutrophils to the location of the inflammation. Finally, the cytokine storm occurs. It is a phenomenon in which uncontrolled large amounts of cytokines are rapidly produced in response to an infectious agent. This unbridled response destroys lung tissue and impairs its function in exchanging respiratory gases.^10,11^

Mesenchymal stems cells (MSCs) are a well-known subset of stem cells recommended to treat COVID- 19.^12^ The expression and secretion of specific cytokines by MSCs may help improve severe viral infection. They possibly can have immunomodulatory properties and a rapid effect on reducing inflammation and tissue damage.^13^ They are promising tools that can suppress the cytokine storm by secreting paracrine or creating a direct interaction with some immune cells.^14,15^ In various clinical trials, MSC therapies have already been shown to be effective and safe. It has been used in some inflammatory diseases mediated by the immune system, such as GVHD and SLE.^16,17^ The effects of the MSCs on the immune system are further elevated by activating the Toll-like receptors (TLRs) in MSCs. These receptors can be stimulated by pathogen-related molecules such as double-strand RNA of various viruses or lipopolysaccharides.^18,19^

Recently some studies have tried to assess the role of MSCs in the modulation of cytokine storms, regulation of Inflammatory response, pulmonary adaptation, preservation of the alveolar microenvironment and endogenous tissue repair. Some studies have proposed the hypothesis of the possible role of MSCs in treating the COVID-19.^20^ The role of MSCs in treating COVID-19 needs to be investigated in different aspects. This brief review aims to investigate the possible healing effect of MSC in COVID-19 and describe the molecular mechanisms by which MSC therapy can probably be beneficial for COVID-19 treatment. This novel method needs to be investigated more since it is cheap, easy to obtain, and without side effects.

## Pathogenesis of COVID-19

According to the WHO, the new coronavirus called SARS-CoV-2 is the agent of the COVID-19 worldwide. The COVID-19 Common symptoms include fever, shortness of breath, and cough. Some of the COVID-19 symptoms that are less common include sore throat, indigestion, redness of the eyes, pain in muscles, and sputum.^21-23^ Most COVID-19 cases show mild symptoms.^24^ However, the severe involvement of some organs is also observed. For example, the disease may lead to respiratory failure of the lungs in a subset of patients. Some other patients may experience chest pains.^25^ In 56.4% of cases, an opaque glass sign was observed on the patients’ chest scan results. However, approximately three percent of severe patients did not exhibit any signs of a problem in their radiology or scintigraphy outcomes.^26,27^ The mortality rate is reported to vary from one to five percent, depending on age and health conditions.^28,29^

Tiny respiratory droplets are considered the leading cause of the disease. The disease can be transmitted when people are infected by patients’ coughs or sneezes.^30^ The duration of exposure to the virus and the onset of symptoms is between two to fourteen days.^27^ The lung is an organ that can be severely affected by COVID-19 due to the abundance of angiotensin-converting enzyme 2 (ACE2) receptors in alveolar type II lung cells. ACE2 is known as the pivotal receptor for virus entry. The virus can enter the cell when a specific type of glycoprotein, called a spike, binds to ACE2.^31-33^ The ACE2 concentration in each tissue is associated with disease severity in that tissue, so it can be assumed that decreasing the ACE2 activity may be a protective strategy in drug repurposing.^34,35^

The new coronavirus may also lead to respiratory failure by impacting the brainstem since other CoVs have been previously discovered to attack the central nervous system (CNS). The virus has been identified in the autopsy of cerebrospinal fluid; the exact mechanism of its pathogenesis in the CNS remains unknown. It possibly attacks the peripheral nerves since low levels of ACE2 are available in the brain.^36,37^ ACE2 is found abundantly in the epithelial and endothelial cells of the gastrointestinal tract organs. The virus also influences the organs of the digestive tract.^38,39^

The virus may lead to acute and chronic damage to cardiovascular system.^40^ However, the acute seems to be more common in patients.^41^ The reason may be ACE2 in myocardial cells that play a role in heart function and is highly expressed in these cells, causing inflammatory responses.^41,42^ Besides, blood vessel function and clot formation play a pivotal role in COVID-19 mortality. The formed clots may lead to pulmonary embolism or ischemic events in the brain. Infection with SARS-CoV-2 can lead to a chain of the vaso-constrictor responses in the body. The narrowing of the blood vessels within the respiratory system circulation has been suggested to reduce oxygen delivery.^43^

Another major cause of death is acute kidney damage. Mainly in people with chronic diseases, including high blood pressure and diabetes, which in particular causes nephropathy in the long term.^44^ The number of lymphocytes, especially natural killer cells, decreases to a lower level in peripheral blood of a subset of COVID-19 patients.^45^ Inflammatory parameters, including C-reactive protein and some cytokines, are overexposed, including *interleukin (*IL)-6, tumor necrosis factor alpha (TNFα), and IL-8.^46,47^ The immune system becomes destroyed by atrophy of lymph nodes and spleen, accompanying a decrease in lymphocytes in the lymph nodes. Most immune cells that penetrate the lung lesions are reported to be macrophages and monocytes. However, the minimum penetration of lymphocytes is reported.^48^ In SARS-CoV-2, Similar to SARS and MERS viral infections, the Cytokine storm and acute respiratory distress syndrome (ARDS) is observed due to excessive secretion of inflammatory factors.^49,50^

**Figure 1:**
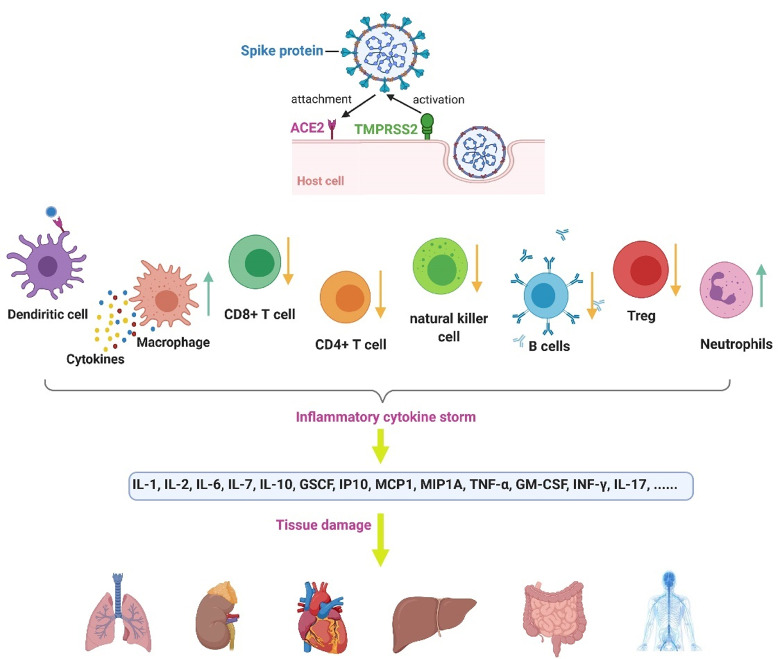
Coronavirus pathogenesis. The viral spike protein becomes activated by TMPRSS2 (a cell protease). The spike then binds to the ACE2 receptor in the host cell.^55^ Infection with COVID-19 causes intracellular components and virus particles and released to the extracellular space, which results in the attraction of immune cells and massive inflammatory responses.^56^ The total count of some lymphocytes, including CD4 ^+^ T cells, CD ^+^ 8 T cells, Treg cells, NK cells, and B cells, decreases. However, the count of neutrophils and leukocytes increases. In COVID-19, the dendritic cells present the antigens.^57^ Besides, the macrophages' vast release of cytokines, which can contribute to the cytokine storm and its followed tissue damages.^58^ The figure was created by BioRender.com

Huang et al investigated the inflammatory factors and cytokine levels in patients with COVID-19. Forty-one patients were hospitalized with various cytokines, including IL-1B, IL-1RA, interleukins (IL-7, 8, 9, and 10), fibroblast growth factor, and granulocyte-mucosal factor. Other cytokines are also reported in severe patients, including MCP1, MIP1A, PDGF, TNFα, GM-CSF, IFNG, G-CSF, IP10, VEGF, IL-2.^51-53^ Choosing a suitable approach to block the cytokine storm and when to use anti-inflammatory medications to reduce COVID-19 mortality is a critical decision.^54^ We have summarized the commonly known pathogenesis of the Coronavirus in Figure 1.

## Stem cell therapy

Cell therapy is based on using the regeneration potential of stem cells to treat some diseases. The therapeutic use of stem cells rehabilitates patients after traumatic injuries in some severe illnesses.^55,56^ Stem cell therapy is described as the usage of stem cells to heal or limit diseases or conditions.^57^ Bone marrow transplantation is the most common treatment for stem cells, but some treatments for umbilical cord blood, adipose stem cells, and embryonic stem cells are also being used.^58^ In recent years, there have been several successful stem cell therapies worldwide. Various stem cells have been used in the treatments, including stem cells isolated from adipose tissue, umbilical cord tissue, and other sources.^58,59^ Research on various stem cell sources is underway to help treat various diseases, including neurological conditions, diabetes, heart disease, respiratory diseases and especially coronavirus infection.^60^ Stem cells have the potential to repair damaged tissue. They have a unique ability to become cells in the blood, liver, myocardium, bone, cartilage or nerve tissue, and thus can repair damaged organs and restore their function.^61^ Immunosuppression is the other use of stem cell therapy.^62^ Inflammation is one of the essential parts of an efficient immune response. Successful removal of infection without causing inflammation is one of the most challenging.^63^ The inflammatory response first starts with the identification of pathogens. Then, the pathogens cause the body’s immune cells to be absorbed into the infected region. The immune cells then destroy the pathogens and eventually lead to homeostasis and tissue repair.^64^ However, SARS-CoV-2 increases the long-term response of cytokines/chemokines in infected patients, called cytokine storms. A cytokine storm leads to ARDS or dysfunction of several organs, and ultimately death. Immediate control of cytokine storm in its preceding stages is the key to successfully treating the patients and decreasing the mortality rate.^65^ Stem cell therapy using different stem cell sources has shown promising cytokine storm regulation.^59,65^

## MSC therapy

MSCs therapy can be useful in two ways. Firstly, it affects and modulates the immune system. Secondly, cell differentiation’s ability possibly contributes to regenerate the damaged tissue to some extent since they can differentiate into various cells. MScs are easy to access since they can be prepared from vast resources such as bone marrow, adipose tissue cells, fetal liver, umbilical cord blood, mobilized peripheral blood, fetal lung, placenta, umbilical cord, tooth pulp, synovial membrane, endometrium, and trabecular.^19,60,66^

MSCs produce a large variety of cytokines by paracrine secretion. Besides, they have direct immunomodulatory activity by direct interaction with various parts of the immune system. MSCs are stimulated by a pathogenic agent, such as lipid polysaccharide or RNA strands of viruses.^18^ The restorative properties of these cells also may enhance endogenous repair.^67^

Some studies have investigated MSCs trafficking and homing. A study of liver resection in mice showed that after transplanting the trackable MSCs in mice tail vein, MSCs first became located in the lungs and then gradually moved to the damaged liver and proliferated. The liver histology and function assessment indicated that the inflammation caused by liver resection subsided after transplanting the MSCs. It can be concluded that MSCs have an immune-regulatory and anti-inflammatory effect. They have a multi-grade differentiation potential.^68,69^ It has been reported that the lung structures such as bronchi or alveoli surfaces remain intact after MSCs are located and trapped in the lungs. It probably is promising and shows that the migration of MSCs into the lungs in their first movement stages does not probably harm the lungs.^70^

When MSCs are injected intravenously, they lead to partial involvement of the lungs. A broad range of mediators mediates the process, including extracellular vesicles, antimicrobial peptides (AMPs), anti-inflammatory cytokines, and secreted angiogenic growth factors.^71,72^ The release pattern of the mediators is regulated by activating pathogen-related receptors. The pathogen-related receptors are expressed in MSCs.^73,74^ TLRs leads to the activation of MSCs and some cell signaling pathways.^18^ For example, TLR3 can be activated by viral RNA. However, TLR9 becomes activated by viral CpF-DNA. MSCs are also reported to impact the repair of alveolar and capillary disorders in the injured lung by secretion of keratinocyte growth factor and Ang-1.^73^

In COVID-19 respiratory viral infection, MSC may also offer two distinct antiviral mechanisms. First, it probably mediates antiviral protection by increasing MSC-specific interferon-stimulated gene (ISG) and secondary response to IFN, leading to ISG induction and widespread viral resistance. MSCs also activate innate and acquired immune systems.^73,75^ MSCs have an effective immune system regulatory activity (immunomodulatory). Based on these reasons, so far, 82 clinical trials have been registered to evaluate the possible therapeutic effect of MSC therapy in treating SARS-CoV-2 infection (available in Table 1)

**Table 1:** List of the registered MSC therapy clinical trials for treating SARS-CoV-2 infection

**No.**	**MSC type**	**Participants number**	**Locations**	**Registration code**
1	Allogenic human cord tissue mesenchymal stromal cells (hCT-MSC)	No.: 30 participants	Duke Hospital, Durham, North Carolina, United States	NCT04399889
2	WJ-MSCs	No.: 5 participants	Stem Cells Arabia, Amman, Jordan	NCT04313322
3	Mesenchymal stem (stromal) cells (MSCs)	No.: 20 participants	Beijing Hospital of China	NCT04252118
4	UC-MSCs	No.: 30 participants	Puren Hospital, Wuhan, Hubei, China	NCT04339660
5	MSCs	No.: 40 participants	University Hospital Tuebingen, Tuebingen, Germany	NCT04377334
6	UC-MSCs	No.: 48 participants	Union Hospital, Wuhan, China	NCT04273646
7	BM-MSCs	No.: 20 participants	Guangzhou Institute of Respiratory Health, China	NCT04346368
8	UC-MSCs	No.: 100 participants	Maternal and Child Hospital of Hubei, China	NCT04288102
9	UC-MSCs	No.: 0 participants (possibly not recruited yet)	Puren Hospital Wuhan, Hubei, China	NCT04293692
10	Remestemcel-L	No.: 50 adult participants	Mount Sinai Hospital, New York, US	NCT04366830
11	Umbilical cord mesenchymal stem cells (UC-MSC)	No.: 24 participants	Diabetes Research Institute, Miami, Florida, US	NCT04355728
12	ACT-20-MSC -umbilical derived mesenchymal stem cells in conditioned media)	No.: 70 participants	Aspire Health Science, United States	NCT04398303
13	Remestemcel-L	No.: 300 participants	-The University of Southern California, -Lutheran Hospital, Fort Wayne, US -Emory University, Atlanta, US, and 12 more.	NCT04371393
14	XCEL-UMC-BETA	No.: 30 participants	-Hospital de Bellvitge, Spain -Mútua de Terrassa, Spain -Hospital del Mar, Spain -and two more	NCT04390139
15	MSC	No.: 60 participants	Royan Institute, Tehran, Iran	NCT04366063
16	Autologous adipose MSC's	No.: 20 participants	Regeneris Medical, United States	NCT04352803
17	MSCs	No.: 30 participants	Houston Methodist Hospital, Houston, Texas, United States	NCT04345601
18	UC-MSCs	No.: 10 participants	Zhongnan Hospital of Wuhan University, China	NCT04269525
19	MSCs	No.: 9 participants	The Ottawa Hospital, Ottawa, Ontario, Canada	NCT04400032
20	Umbilical cord-derived MSCs	No.: 75 participants	Northern Ireland, United Kingdom	NCT03042143
21	MSC treatment	No.: 30 participants	Istinye University, Istanbul, Turkey; SBÜ Dr. Sadi Konuk Eğitim ve Araştırma Hastanesi, Istanbul, Turkey.	NCT04392778
22	Bone marrow harvest	No.: 10 participants	Cambridge University Hospitals NHS	NCT04397471
23	Umbilical cord Wharton's jelly-derived human	No.: 40 participants	Hôpital Pitié-Salpêtrière – APHP, Paris, France; Hôpital Européen Georges Pompidou – APHP, Paris, France	NCT04333368
24	Wharton's jelly derived MSCs + standard therapy	No.: 40 participants	BioXcellerator, Medellin, Antioquia-CO, Colombia; Clinical Somer, Rionegro, Antioquia, Colombia	NCT04390152
25	BM-Allo.MSC	No.: 45 participants	St. Francis Medical Center, Lynwood, California, United States	NCT04397796
26	MSCs-derived exosomes	No.: 30 participants	Ruijin Hospital Shanghai Jiao Tong University School of Medicine, Shanghai, Shanghai, China	NCT04276987
27	NK cells and MSCs	No.: 20 participants	Hospital of Nanchang University, China	ChiCTR2000030944
28	HUMSCs and exosomes treat	No.: 90 participants	Shiyan Taihe hospital, China	ChiCTR2000030484
29	MSCs	No.: 20 participants	Hospital of Xinxiang Medical University, China	ChiCTR2000030835
30	UC-MSC	No.: 30 participants Control group:30	Chinese PLA General Hospital, China	ChiCTR2000030138
31	Human umbilical cord MSCs	No.: 30 participants	The First Hospital of Changsha, China	ChiCTR2000030866
32	Human umbilical cord MSCs	No.: 100 participants Control group:100	The Fifth Medical Center of PLA General Hospital, China	ChiCTR2000031430
33	HB-adMSCs	No.: 100 participants	Hope Biosciences Stem Cell Research Foundation, Sugar Land, Texas, United States	NCT04348435
34	HB-adMSCs	No.: 56 participants	Hope Biosciences Stem Cell Research Foundation, Sugar Land, Texas, United States	NCT04349631
35	MSCs	No.: 60 participants	Royan Institute, Iran	IRCT20200217046526N2
36	Placental MSCs	No.: 20 participants	Tarbiat Modares University, Iran	IRCT20200413047063N1
37	HB-adMSC	No.: 100 participants	River Oaks Hospital and Clinics, Houston, Texas, United States; United Memorial Medical Center, Houston, Texas, United States	NCT04362189
38	Mesenchymoangioblast-derived MSCs	No.: 24 participants	Cynata Therapeutics Limited, Australia	ACTRN12620000612910
39	MSCs	No.: 6 participants	Royan Institute, Iran	IRCT20200217046526N1
40	Allogenic mesenchymal stem cell-derived umbilical cord transplantation	No.: 20 participants	Mashhad University of Medical Sciences, Iran	IRCT20160809029275N1
41	MSCT	No.: 150 participants	Assiut university, Assiut, Egypt	NCT04492865
42	P-MMSCs	No.: 30 participants	Institute of Cell Therapy, Kyiv, Ukraine	NCT04461925
43	MSC	No.: 20 participants	Hospital Regional Lic Adolfo Lopez Mateos, Mexico City, Ciudad De Mexico CDMX (Mexico City), Mexico	NCT04611256
44	LMSCs	No.: 70 participants	Miami VA Healthcare System, Miami, Florida, United States; University of Maryland Medical Center, Baltimore, Maryland, United States; Wake Forest Baptist Medical Center, Winston-Salem, North Carolina, United States	NCT04629105
45	UCMSCs	No.: 21 participants	University of Miami, Miami, Florida, United States	NCT04490486
46	Mesenchymal stromal stem cells - KI-MSC-PL-205	No.: 9 participants	Uppsala University Hospital, Uppsala, Sweden	NCT04447833
47	MSC	No.: 10 participants	Royal Perth Hospital Cell & Tissue Therapies WA, Australia	ACTRN12620000840987
48	Human umbilical cord MSCs	No.: 16 participants	The First Affiliated Hospital of Nanchang University, China	ChiCTR2000030116
49	hucMSCs	No.: 9 participants	Nanjing Second Hospital, China	ChiCTR2000030300
50	umbilical cord MSCs	No.: 14 Control: 14 Exp	Histocell S.L., Spain	EUCTR2019-002688-89-ES
51	Human umbilical cord MSCs	No.: 0 participants (possibly not recruited yet)	CHU de Liège, Belgium	EUCTR2020-002102-58-BE
52	Remestemcel-L	No.: 0 participants (possibly not recruited yet)	Hospital Universitario Puerta de Hierro, Spain	EUCTR2020-002193-27-ES
53	MSC	No.: 50 Control, 50 Exp	Fundación Instituto de Investigación Sanitaria Fundación Jiménez Díaz, Spain	EUCTR2020-001266-11-ES
54	Human umbilical cord MSCs	No.: 15 Control, 15 Exp	Banc de Sang i Teixits, Spain	EUCTR2020-001505-22-ES
55	MSC	No.: 5 participants	Bagheiat-allah University of Medical Sciences, Iran	IRCT20200325046860N2
56	MSC	No.: 30 participants	Hamedan University of Medical Sciences, Iran	IRCT20200426047206N2
57	Human umbilical cord MSCs	No.: 45 Control, 45 Exp	Shahid Modares hospital, Iran	IRCT20200421047150N1
58	Adipose-derived MSCs	No.: 13 control, 13 Exp	Hospital Universitario de Jerez de la Frontera, Jerez de la Frontera, Cádiz, Spain; Hospital Reina Sofía, Córdoba, Spain; Hospital Universitario Virgen de las Nieves, Granada, Spain (and 3 more...)	NCT04366323
59	Human umbilical cord MSCs	No.: 15 Control 15 Exp	-Fundación Universitaria de Ciencias de la Salud. -Hospital de San Jose. -Hospital Infantil Universitario de San Jose, Colombia	NCT04429763
60	AdMSCs	No.: 100 Control, 100 Exp	Celltex Therapeutics Corporation, United States	NCT04428801
61	MSCs	No.: 5 Control, 5 Exp	Instituto Nacional de Ciencias Médicas y Nutrición Salvador Zubirán, Mexico City, Mexico	NCT04416139
62	Pooled olfactory mucosa-derived MScs	No.: 20 Control, 20 Exp	Institute of Biophysics and Cell Engineering of National Academy of Sciences of Belarus, Minsk, Belarus	NCT04382547
63	Remestemcel-L	No.: 53 Control, 53 Exp	Hospital Universitario de Getafe, Getafe, Madrid, Spain; Hospital Universitario de Cruces, Barakaldo, Spain; Hospital Universitario de La Princesa, Madrid, Spain (and 3 more...)	NCT04366271
64	Remestemcel-L	No.: 30 Control, 30 Exp	Fuzhou General Hospital, Fuzhou, Fujian, China	NCT04371601
65	Remestemcel-L	No.: 12 Control, 12 Exp	Hospital Universitario Rio Hortega, Valladolid, Spain	NCT04361942
66	Remestemcel-L	No.: 13 Control, 13 Exp	Red Andaluza de Diseño y Traslación de Terapias Avanzadas-Fundación Progreso y Salud, Spain	EUCTR2020-001364-29-ES
67	Human umbilical cord MSCs	No.: 10 Exp	Iran University Of Medical Science, Iran	IRCT20140528017891N8
68	Dental mesenchymal pulp stem cells	No.: 10 Exp	Kerman University of Medical Sciences, Iran	IRCT20140911019125N6
69	Remestemcel-L	No.: 12 Control, 12 Exp	CITOSPIN S.L., Spain	EUCTR2020-001682-36-ES
70	Adipose-derived MSCs	No.: 50 Control, 50 Exp	-Instituto de Investigación Sanitaria de la Fundación Jiménez Díaz. -Instituto de Investigación Sanitaria y Biomédica de Alicante. -Hospital General Universitario Gregorio Marañon. -Clinica Universidad de Navarra, Universidad de Navarra. -Hospital Universitario de Salamanca. -Hospital General Universitario de Alicante. -Hospital Clínico Universitario Virgen de la Arrixaca. Spain	NCT04348461
71	Adipose-derived MScs	No.: 20 Control, 20 Exp	2014 Department of Cardiology, The Heart Centre, University Hospital, Rigshospitalet, Copenhagen, Denmark	NCT04341610
72	Dental mesenchymal pulp stem cells	No.: 10 Control, 10 Exp	Renmin Hospital of Wuhan University (East Campus), Wuhan, Hubei, China	NCT04336254
73	Remestemcel-L	No.: 66 Exp	Hospital Vera Cruz, Campinas, São Paulo, Brazil; Hospital de Barueri, São Paulo, Brazil; IncCOR, São Paulo, Brazil; UNIFESP, São Paulo, Brazil	NCT04315987
74	Remestemcel-L	No.: 18 Control, 18 Exp	The Jiangsu Cell Tech Medical Research Institute, China	ChiCTR2000031494
75	Dental mesenchymal pulp stem cells	No.: 10 Control, 10 Exp	Center for Regenerative Medicine, Renmin Hospital of Wuhan University, China	ChiCTR2000031319
76	Dental mesenchymal pulp stem cells	No.: 24 Exp	CAR-T (Shanghai) Biotechnology Co., Ltd., China	NCT04302519
77	MSCs (origin not specified) + Ruxolitinib	No.: 35 Control, 35 Exp	Department of Hematology, Tongji Hospital, Tongji Medical College, Huazhong University of Science and Technology, China	ChiCTR2000029580
78	MSC exosomes (origin not specified)	No.: 13 Control, 13 Exp	Wuxi Fifth People's Hospital, China	ChiCTR2000030261
79	Remestemcel-L	No.: 20 Exp	Second Hospital of University of South China, Hengyang, China	ChiCTR2000030020
80	Human umbilical cord MSCs	No.: 20 Control, 20 Exp	The Sixth Medical Center of PLA General Hospital, China	ChiCTR2000030088
81	Remestemcel-L	No.: 60 Control, 60 Exp	Institute of Basic Medical Sciences, Chinese Academy of Medical Sciences, China	ChiCTR2000029990
82	Human umbilical cord MSCs	No.: 30 Control, 30 Exp	Hunan yuanpin Cell Biotechnology Co., Ltd, China	ChiCTR2000030173

**Figure 2:**
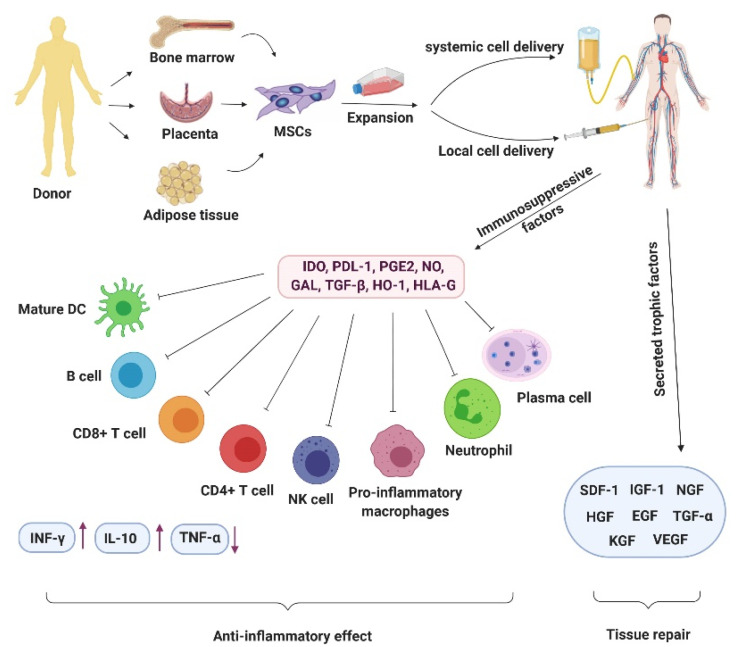
The picture describes the crucial molecules and cells intermediating in MSC therapy in COVID-19 patients. The figure was created by BioRender.com

A study at Beijing’s YouAn hospital examined whether the treatment using MSC improved the outcomes of seven patients admitted with COVID-19 pneumonia. Various clinical results of MSC injection were investigated in seven patients within 14 days. The clinical results included changes in inflammation, immune function, and side effects. They reported that MSCs improved the performance of seven patients without observation of any side effects. Pulmonary function and manifestations of these seven patients improved significantly during two days after MSC treatment. Peripheral lymphocytes were then extracted and studied. The C-reactive protein was reduced. Inactive cytokine-secreting immune cells vanished within three to six days, including CXCR3+ CD8+ T cells, CXCR3+ CD4+ T cells, and CXCR3+ NK cells. Besides, a subset of DC cell populations regulating CD14+ CD11c+ CD11bmid increased significantly. TNF-α levels were decreased significantly. However, IL-10 levels augmented in the MSC therapy group compared with the placebo. The gene expression specifications indicated that MSCs were negative for TMPRSS2 and ACE2, showing that MSCs were probably free of the SARS-CoV-2. Capillary endothelial and alveolar (type II) cells can express the ACE2 and TMPRSS2 receptors. These receptors can help the virus enter into the host cell and contribute to its spreading.^73,76^ In sum, they reported that intravenous transplantation of MSCs was useful and safe in the treatment of a subset of patients with COVID-19.^76^ We have summarized the crucial molecules and cells intermediating in MSC therapy in COVID-19 patients in Figure 2.

## Secretome of MSCs in COVID-19 therapy

At the beginning of the viral disease, pneumocytes type II are infected; therefore, the respiratory system becomes injured.^77^ Previous studies have shown that the secretome of MSCs can be applied to treat injuries in the respiratory system. The MSC secretome has therapeutic ingredients, including exosomes, microvesicles, various proteins, cytokines, miRNAs, and lipids.^77^ The exosomes derived from the MSCs of the bone marrow improve lung oxygenation. The exosomes are shown to modulate lymphocyte counts and reduce cytokine storms. In sum, it could improve the health of hospitalized COVID-19 patients.^78^ Bari et al have shown that the secretome could be a useful therapeutic in the pneumonia of COVID-19 patients.^78^ The secretome modulates the immune system and has an anti-inflammatory function. It also has an essential role in pro-angiogenic and anti-fibrotic processes.^79^

MSCs have potential antimicrobial impacts and can directly or indirectly stimulate the immune system since they can produce some AMPs when pathogens infect the organism.^80^ They can probably be considered a first-line defense that protects the organism against various infections.^81^ AMPs may have extracellular or intracellular molecular targets; therefore, they can disrupt membrane integrity or inhibit proteins or DNA/RNA synthesis. MSCs produce various AMP types, such as the human cysteine-rich protein called β-defensin (hBD). The hBD contributes to the human body in defending against microbes. MSCs and some other cells can secrete different β-Defensin family members such as hBD-1, hBD-2 and hBD-3.^82-84^ Defensin can inhibit some viral cycle mechanisms. For example, it can inhibit the viral entry and traffic against the respiratory syncytial virus, influenza A virus, and some coronaviruses (SARS). This therapeutic effect of defensin can boost the host immune system and lead to dysfunction of the infectious pathogens. It also can contribute to employ various immune cells such as dendrites, T cells, and macrophages into some tissues.^85^ Some AMPs can facilitate bacterial clearance during MSC therapy and even contribute to ARDS inhibition.^86,87^ Finally, it has been hypothesized that the use of MSCs’ secretome can be an alternative method in treating COVID-19 patients in their severe condition.

## Conclusion

The safety and anti-inflammatory properties of MSC therapy in the treatment of COVID-19 have been confirmed and documented by 40 clinical studies (https://clinicaltrials.gov) (https://www.who.int/ictrp/en/). MSC therapy seems to be promising in patients with COVID-19. The essential sources of MSCs include the umbilical cord, umbilical cord blood, Wharton gel, menstrual blood, and bone marrow. The therapeutic results are expected to be observed in a short period. We suggest a combination of therapeutic approaches for patients with COVID-19. However, we emphasize that MSC therapy should be more paid attention to and investigated in this regard. Further scientific investigations probably will ensure the effectiveness and safety of this type of treatment soon.

## Acknowledgments

We thank the Proteomics Research Centre of Shahid Beheshti University of Medical Sciences for their support in this study.

## Ethical Issues

The ethics committee of Shahid Beheshti University of Medical Sciences approved this study.

## Conflict of Interest

The authors declare that they have no conflict of interests.
